# Association between NUTRIC score and ICU mortality in patients with sepsis: a prospective cohort study

**DOI:** 10.3389/fnut.2025.1654901

**Published:** 2025-07-31

**Authors:** Mingjie Xie, Liuyun Huang, Ling Li, Yuanyuan Qin, Biheng Feng, Qingjiang Cai, Debin Huang

**Affiliations:** ^1^Department of Critical Care Medicine, The First Affiliated Hospital of Guangxi Medical University, Nanning, China; ^2^Guangxi Clinical Research Center for Critical Care Medicine, Nanning, China

**Keywords:** sepsis, nutrition risk in critically ill score, NUTRIC score, ICU mortality, cohort study

## Abstract

**Purpose:**

This study aimed to explore the association between the Nutrition Risk in Critical Illness (NUTRIC) score and the risk of ICU mortality in patients with sepsis.

**Methods:**

This was a single-center, prospective cohort study that enrolled septic patients admitted between November 2024 and May 2025 to Wards 1 and 2 of the Department of Critical Care Medicine at the First Affiliated Hospital of Guangxi Medical University. A multivariable logistic regression model was applied to evaluate the association between the NUTRIC score assessed within 24 h of ICU admission and ICU mortality. Restricted cubic spline (RCS) analysis was conducted to model this relationship, and robustness was verified via subgroup analysis. Kaplan–Meier survival curve analysis was used to compare cumulative ICU survival rates among different NUTRIC score groups, with differences between groups tested using the log-rank test.

**Results:**

A total of 245 patients with sepsis were included in the study, and the ICU mortality rate was 17.1% (42/245). Multivariable logistic regression showed a statistically significant association between the NUTRIC score and ICU mortality, with each 1-point increase in the score associated with a 92% increase in risk (OR = 1.92, 95% CI: 1.32–2.80, *p* = 0.002). RCS analysis indicated a significant linear relationship between the NUTRIC score and ICU mortality risk (P _non-linearity_ = 0.704). Subgroup analysis further demonstrated a positive association across all subgroups (ORs > 1), and did not identify any significant interactions. Kaplan–Meier survival curves showed that patients with high nutritional risk (NUTRICb group) had significantly poorer ICU survival than those with low nutritional risk (NUTRICa group) (log-rank test, *p* = 0.00024).

**Conclusion:**

The NUTRIC score is significantly associated with ICU mortality in patients with sepsis, highlighting its potential utility in early ICU risk stratification. Incorporating the NUTRIC score into ICU assessment protocols may help identify high-risk patients early and guide early nutrition or supportive care, potentially improving clinical outcomes.

## 1 Introduction

Malnutrition is a significant public health issue worldwide. In the intensive care unit (ICU), critically ill patients often experience metabolic stress, characterized by hypermetabolism, excessive release of inflammatory cytokines, and progressive gastrointestinal dysfunction, which further exacerbates the depletion of nutrients. Studies (1–3) have shown that the incidence of malnutrition in critically ill patients is 40–80% higher than in general hospitalized patients, with approximately 38–78% of patients being malnourished upon ICU admission. According to the guidelines (4) from the European Society for Clinical Nutrition and Metabolism (ESPEN), all ICU patients who stay for more than 48 h are at risk of malnutrition. Sepsis is one of the most common critical illnesses in the ICU, often resulting in a hypermetabolic state due to an excessive acute-phase response and gastrointestinal dysfunction. This metabolic abnormality is further exacerbated by the cascading activation of inflammatory mediators, significantly increasing the risk of malnutrition (5).

The Society of Critical Care Medicine (SCCM) and the American Society for Parenteral and Enteral Nutrition (A. S. P. E. N.) recommend the use of nutritional risk screening tools, including the Nutritional Risk Screening 2002 (NRS-2002), the Nutrition Risk in Critical Illness(NUTRIC) score, and the modified NUTRIC(mNUTRIC) score, for early nutritional risk assessment in patients (6). Compared to other commonly used nutritional risk screening tools, the NUTRIC score, specifically designed for ICU patients, demonstrates superior identification ability and more effectively identifies patients who are most likely to benefit from nutritional intervention (7).

Nutritional risk assessment plays a crucial role in identifying high-risk patients and guiding targeted therapeutic and nursing strategies, thereby improving clinical outcomes. In sepsis patients, a hypermetabolic state and insufficient nutritional intake contribute to a negative nitrogen balance, which is significantly associated with higher mortality rates in the ICU (8). In recent years, with the growing demand for intensive care and the advancement of evidence-based medicine, the importance of precise nutritional assessment tools in optimizing ICU resource allocation and improving patient prognosis has become increasingly evident (9). However, to the best of our knowledge, no study has specifically assessed the correlation between the NUTRIC score and ICU mortality risk in sepsis patients. Therefore, we conducted a single-center, prospective cohort study aimed at exploring the association between the NUTRIC score and ICU mortality in sepsis patients, to provide evidence-based support for critical care nutrition therapy and improve the prognosis of sepsis patients in the ICU.

## 2 Population and methods

### 2.1 Study design, setting and population

**Figure 1:**
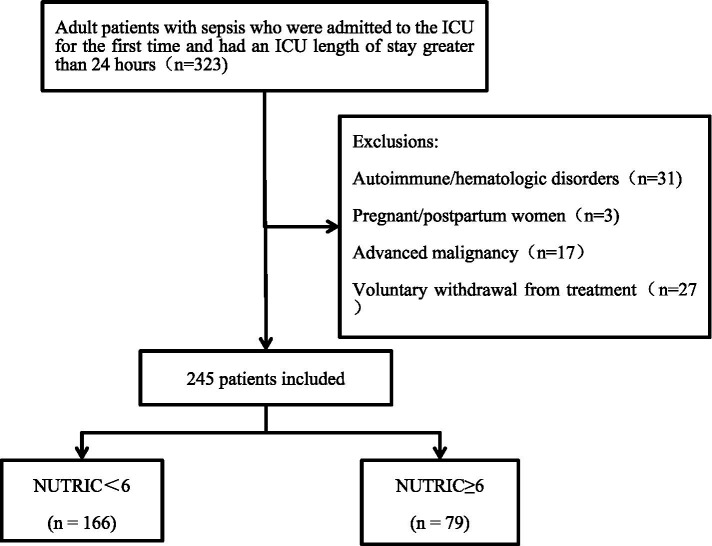
Flowchart of patient enrollment and exclusion.

This was an observational, prospective, single-center study conducted between November 2024 and May 2025 in Wards 1 and 2 of the Department of Critical Care Medicine, First Affiliated Hospital of Guangxi Medical University. A total of 323 adult sepsis patients whose ICU stay exceeded 24 h were initially screened. To ensure that nutritional status was predominantly influenced by the acute septic episode rather than by confounding conditions, we excluded patients with autoimmune diseases, hematologic disorders, pregnancy, advanced malignancies, or those who withdrew consent (*n* = 78). These conditions were omitted because they can independently alter nutritional risk and IL-6 levels through hormonal fluctuations, chronic inflammation, or cachexia, thereby potentially biasing the NUTRIC score. After these exclusions, 245 patients were retained for final analysis. Nutritional risk was assessed within 24 h of ICU admission using the NUTRIC score (7): NUTRICa (NUTRIC <6) and NUTRICb (NUTRIC ≥6). The patient selection process is detailed in Figure 1.

The study protocol was reviewed and approved by the Ethics Committee of the First Affiliated Hospital of Guangxi Medical University (approval number: 2025-K0138). All study procedures were performed in compliance with the ethical standards set forth in the Declaration of Helsinki, ensuring research integrity and the protection of participant welfare. The study design and reporting conform to the STROBE guidelines (10) for observational research.

### 2.2 Data collection

Patient data were extracted from the electronic medical records system and included the following variables: (1) demographic and baseline characteristics: gender, age, smoking history, alcohol consumption history, and pre-ICU hospital length of stay; (2) comorbidities: coronary artery disease, diabetes, and hypertension; (3) complications: septic shock, acute kidney injury (AKI), multiple organ dysfunction syndrome (MODS), and acute respiratory distress syndrome (ARDS); (4) clinical scores: Acute Physiology and Chronic Health Evaluation II (APACHE II), Sequential Organ Failure Assessment (SOFA), Charlson Comorbidity Index (CCI), and shock index; (5) laboratory parameters within 24 h of ICU admission: interleukin-6 (IL-6), alanine aminotransferase (ALT), aspartate aminotransferase (AST), blood glucose(Glu), creatinine (Cr), hemoglobin (Hb), international normalized ratio (INR), serum potassium (K^+^), serum sodium (Na^+^), lactate (LAC), lymphocyte count (LYM), prealbumin (PA), white blood cell count (WBC), and neutrophil count (NEU); (6) ICU treatment duration: continuous renal replacement therapy (CRRT) and mechanical ventilation; (7) duration of medication use: Neuromuscular Blocking Agents(NMBAs) and vasoactive drugs; (8) primary outcome: ICU mortality rate; (9) secondary outcomes:length of stay in ICU (LOS in ICU) and length of stay in hospital(LOS in hospital).

### 2.3 Definition of sepsis

Sepsis was diagnosed according to the criteria (11) established by the Third International Consensus Definitions for Sepsis and Septic Shock (Sepsis-3), published by the European Society of Intensive Care Medicine in 2016. Sepsis was defined as life-threatening organ dysfunction caused by a dysregulated host response to infection, confirmed by an increase of ≥2 points in the SOFA score from baseline.

### 2.4 NUTRIC score

The NUTRIC score consists of six components: age, APACHE II score, SOFA score, number of comorbidities, pre-ICU hospital length of stay, and IL-6 level. Points are assigned based on predefined ranges for each parameter, and the total score is calculated by summing all component scores. The maximum NUTRIC score is 10 points, and the scoring criteria are summarized in Table 1.

**Table 1:** Evaluation criteria of the NUTRIC score.

Variable	Range	Points
Age(years)	<50	0
	50–74	1
	≥75	2
APACHEII (points)	<15	0
	15–19	1
	20–27	2
	≥28	3
SOFA (points)	<6	0
	6–9	1
	≥10	2
Comorbidities (*n*)	0–1	0
	≥2	1
Pre-ICU hospital length of stay (days)	0–1	0
	>1	1
IL-6 (pg/mL)	<400	0
	≥400	1
		0–10

Two trained researchers independently assessed the nutritional risk of each patient within 24 h of ICU admission using the NUTRIC scoring tool. After completing the evaluations, the two scorers recorded their respective results and compared them. In cases of discrepancy, the final score was determined through discussion and review of the training materials. If necessary, a third trained researcher was consulted to adjudicate and ensure scoring accuracy.

All NUTRIC scores were recorded in a pre-designed data collection form, which included the time of assessment, assessor identity, individual score components, and total score. Data were entered in real time into a secure electronic database by designated personnel responsible for data entry and management. Double data entry and verification were conducted to prevent input errors. Data quality control was performed regularly, including assessment of inter-rater reliability and data completeness.

### 2.5 Statistical analysis

All statistical analyses were performed using R software (version 4.2.2; The R Foundation for Statistical Computing, Vienna, Austria; https://www.r-project.org). The normality of continuous variables was assessed using the Kolmogorov–Smirnov test, in combination with visual inspection of histograms and Q–Q plots. Normally distributed continuous data were presented as mean ± standard deviation (SD), and differences between groups were analyzed using the independent samples *t*-test. Non-normally distributed data were expressed as median and interquartile range [M (P_25_, P_75_)], and compared using the Mann–Whitney *U* test. Categorical variables were presented as frequencies and percentages, and intergroup comparisons were performed using the chi-square test or Fisher’s exact test, as appropriate.

To assess the association between nutritional risk and clinical outcomes, we performed the following analyses: First, multivariable logistic regression analysis was performed to evaluate the association between NUTRIC score and ICU mortality in sepsis patients, with adjustment for potential confounders. Second, restricted cubic spline (RCS) regression was used to examine the dose–response relationship between NUTRIC score and ICU mortality. Third, subgroup analyses were conducted to assess the consistency of this association across different strata, with interaction tests performed to identify potential effect modifiers. Additionally, Kaplan–Meier survival curves were plotted to compare cumulative survival probabilities between high- and low-nutritional-risk groups, with differences assessed using the log-rank test. A two-sided *p*-value <0.05 was considered statistically significant for all analyses.

## 3 Results

### 3.1 Patient characteristics

Among the 245 enrolled sepsis patients, the ICU mortality rate was 17.1% (42/245). Baseline characteristics are presented in Table 2. The cohort had a mean age of 61.3 ± 16.1 years, with 73.5% (180/245) being male and 26.5% (65/245) female. Patients in the high nutritional risk group (NUTRICb) were significantly older, had higher prevalence of hypertension, diabetes, and coronary artery disease, and exhibited higher APACHE II, SOFA, and CCI scores, indicating more severe illness at ICU admission. Moreover, compared with the low nutritional risk group (NUTRICa), NUTRICb patients required significantly longer durations of continuous CRRT and mechanical ventilation.

**Table 2:** Baseline characteristics of patients stratified by NUTRIC score.

Variables	Total (*n* = 245)	NUTRICa (*n* = 166)	NUTRICb (*n* = 79)	Statistic	*p*
Age, years	61.3 ± 16.1	56.0 ± 15.4	72.5 ± 11.1	72.288	**< 0.001**
Gender				0.368	0.544
Male	180 (73.5)	120 (72.3)	60 (75.9)		
Female	65 (26.5)	46 (27.7)	19 (24.1)		
Smoking history	100 (40.8)	66 (39.8)	34 (43)	0.238	0.625
Alcohol consumption history	104 (42.4)	71 (42.8)	33 (41.8)	0.022	0.882
Hypertension	114 (46.5)	63 (38)	51 (64.6)	15.228	**< 0.001**
Diabetes	57 (23.3)	31 (18.7)	26 (32.9)	6.077	**0.014**
Coronary artery disease	28 (11.4)	12 (7.2)	16 (20.3)	8.97	**0.003**
Shock idex	0.9 ± 0.3	0.9 ± 0.3	0.9 ± 0.3	0.855	0.356
Septic shock	198 (80.8)	128 (77.1)	70 (88.6)	4.565	**0.033**
ARDS	69 (28.2)	43 (25.9)	26 (32.9)	1.299	0.254
AKI	86 (35.1)	48 (28.9)	38 (48.1)	8.649	**0.003**
MODS	109 (44.5)	62 (37.3)	47 (59.5)	10.628	**0.001**
pre-ICU hospital length of stay, days	11.0 (4.0, 22.0)	10.0 (3.2, 20.0)	14.0 (4.0, 26.5)	2.955	0.086
APACHEII, points	23.5 ± 7.8	20.4 ± 6.4	30.1 ± 6.3	121.741	**< 0.001**
SOFA, points	6.7 ± 3.6	5.5 ± 2.6	9.3 ± 4.0	81.585	**< 0.001**
CCI, points	6.2 ± 4.0	5.7 ± 3.6	7.5 ± 4.5	11.263	**< 0.001**
WBC,10^9^/L	11.8 (8.6, 17.0)	11.8 (8.7, 17.2)	11.4 (8.1, 16.9)	0.003	0.956
Hb, g/dL	87.3 ± 25.9	87.8 ± 25.9	86.0 ± 26.1	0.271	0.603
LYM, 10^9^/L	0.7 (0.4, 1.3)	0.7 (0.4, 1.3)	0.7 (0.3, 1.2)	0.247	0.619
NEU, × 10^9^/L	9.7 (6.3, 14.4)	9.7 (6.5, 15.0)	9.6 (6.0, 14.4)	0.314	0.575
AST, U/L	44.0 (25.0, 111.0)	42.5 (25.0, 109.5)	55.0 (26.5, 113.5)	1.144	0.285
PA, mg/L	100.9 (66.4, 156.2)	106.6 (65.3, 158.6)	92.1 (69.8, 144.4)	0.675	0.411
ALT, U/L	29.0 (15.0, 71.0)	32.0 (15.0, 72.0)	27.0 (14.5, 59.0)	0.688	0.407
Cr, μmol/L	138.0 (74.0, 301.0)	129.0 (67.2, 289.2)	164.0 (106.0, 384.5)	5.325	**0.021**
INR	1.3 (1.2, 1.6)	1.3 (1.1, 1.6)	1.3 (1.2, 1.5)	0.266	0.606
Glu, mmol/L	12.0 ± 4.8	11.8 ± 4.4	12.3 ± 5.6	0.743	0.389
IL-6, pg./mL	148.0 (47.4, 478.0)	151.5 (44.7, 445.5)	129.0 (64.2, 566.0)	1.745	0.186
Na^+^, mmol/L	140.4 ± 9.6	139.6 ± 9.5	141.9 ± 9.7	3.081	0.08
K^+^, mmol/L	4.0 ± 0.9	4.0 ± 0.9	4.0 ± 0.9	0.022	0.883
LAC, mmol/L	2.1 (1.3, 3.5)	2.0 (1.2, 3.0)	2.5 (1.4, 4.6)	3.812	0.051
Mechanical ventilation, days	7.0 (3.0, 14.0)	7.0 (2.0, 12.8)	9.0 (4.0, 15.0)	5.314	**0.021**
Use of NMBAs, days	2.0 (0.0, 5.0)	2.0 (0.0, 5.0)	2.0 (1.0, 4.0)	0.034	0.854
Use of vasoactive drugs, days	5.0 (2.0, 10.0)	4.0 (2.0, 9.0)	7.0 (3.0, 11.5)	7.693	**0.006**
CRRT, days	0.0 (0.0, 6.0)	0.0 (0.0, 4.0)	3.0 (0.0, 8.0)	16.003	**< 0.001**
LOS in hospital, days	18.0 (10.0, 31.0)	19.0 (11.0, 32.8)	16.0 (9.0, 28.5)	1.943	0.163
LOS in ICU, days	9.0 (5.0, 18.0)	9.0 (5.0, 17.8)	10.0 (6.0, 17.5)	0.191	0.662
ICU mortality	42 (17.1)	16 (9.6)	26 (32.9)	20.411	**< 0.001**

^*^Significant *P*-values are shown in bold.

### 3.2 Association between NUTRIC score and ICU mortality in patients with sepsis

We constructed four multivariate logistic regression models to examine the association between NUTRIC score and ICU mortality (Table 3). Model 1 was unadjusted. Model 2 adjusted for demographic variables (gender, alcohol consumption history, smoking history) and medical history (diabetes, hypertension, coronary artery disease). Model 3 additionally incorporated illness severity parameters (shock index, CCI) and complications (AKI, ARDS, MODS, septic shock). Model 4 further adjusted for laboratory parameters (ALT, AST, Glu, Cr, Hb, INR, Na^+^, K^+^, LAC, LYM, PA, WBC, NEU), treatment durations (CRRT, mechanical ventilation), and medication use (NMBAs, vasoactive drugs).

**Table 3:** Multivariate logistic regression analysis of the association between NUTRIC Score and ICU mortality.

Variable	Model 1		Model 2		Model 3		Model 4	
	OR (95%CI)	*P*-value	OR (95%CI)	*P*-value	OR (95%CI)	*P*-value	OR (95%CI)	*P*-value
NUTRIC	1.80 (1.42 ~ 2.28)	**<0.001**	2.04 (1.5 ~ 2.77)	**<0.001**	1.83 (1.31 ~ 2.54)	**<0.001**	1.92 (1.32 ~ 2.80)	**0.001**
NUTRIC1	1 (Reference)		1 (Reference)		1 (Reference)		1 (Reference)	
NUTRIC2	2.24 (0.39 ~ 12.76)	0.364	2.15 (0.37 ~ 12.5)	0.394	1.66 (0.28 ~ 9.98)	0.578	1.38 (0.19 ~ 9.92)	0.747
NUTRIC3	6.36 (1.33 ~ 30.55)	**0.021**	6.86 (1.37 ~ 34.39)	**0.019**	5.93 (1.12 ~ 31.49)	**0.037**	5.53 (0.92 ~ 33.17)	0.061
NUTRIC4	13.74 (3.11 ~ 60.73)	**0.001**	17.84 (3.43 ~ 92.88)	**0.001**	13.27 (2.31 ~ 76.29)	**0.004**	12.08 (1.8 ~ 81.04)	**0.01**
Trend test	2.40 (1.64 ~ 3.52)	**<0.001**	2.72 (1.69 ~ 4.36)	**<0.001**	2.52 (1.49 ~ 4.25)	**0.001**	2.52 (1.42 ~ 4.47)	**0.002**

^*^Significant *P*-values are shown in bold.

When treated as a continuous variable, the NUTRIC score was identified as a significant risk factor for ICU mortality in patients with sepsis in the unadjusted Model 1, showing a positive association(OR = 1.80, 95% CI:1.42–2.28, *p* < 0.001). This significant positive association persisted across the stepwise adjusted models: Model 2 (OR = 2.04, 95% CI:1.5–2.77, *p* < 0.001), Model 3 (OR = 1.83, 95% CI:1.31–2.54, *p* < 0.001), and the fully adjusted Model 4 (OR = 1.92, 95% CI:1.32–2.80, *p* = 0.001). In Model 4, each one-point increase in the NUTRIC score was associated with a 92% higher risk of ICU mortality among patients with sepsis.

Patients were stratified into quartile-based groups according to their NUTRIC scores for further analysis. In the fully adjusted Model 4, using the lowest NUTRIC quartile (NUTRIC 1) as the reference group, the risk of ICU mortality increased progressively with higher NUTRIC quartiles. This upward trend in ICU mortality risk across increasing NUTRIC quartiles remained statistically significant in all models (Model 1 through Model 4), with trend test *p*-values all < 0.05.

### 3.3 Restricted cubic spline analysis between NUTRIC score and ICU mortality

**Figure 2:**
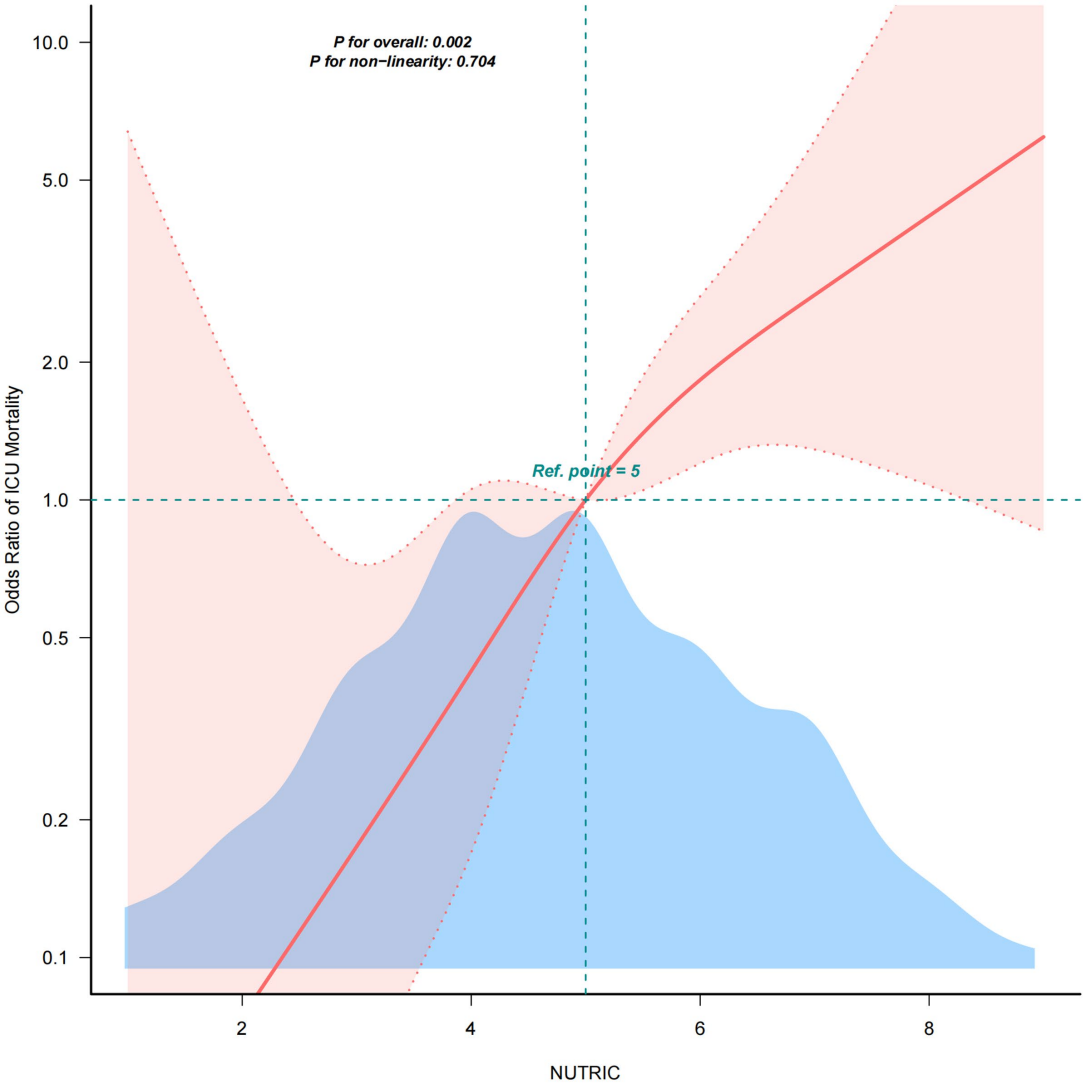
RCS analysis between NUTRIC Score and ICU mortality.

After full adjustment for covariates, RCS analysis was employed to evaluate the dose–response relationship between NUTRIC score and ICU mortality in sepsis patients. As demonstrated in Figure 2, we observed a significant linear association between NUTRIC score and ICU mortality (P _non-linearity_ = 0.704), indicating a progressive increase in mortality risk with higher NUTRIC scores.

### 3.4 Subgroup analysis

**Figure 3:**
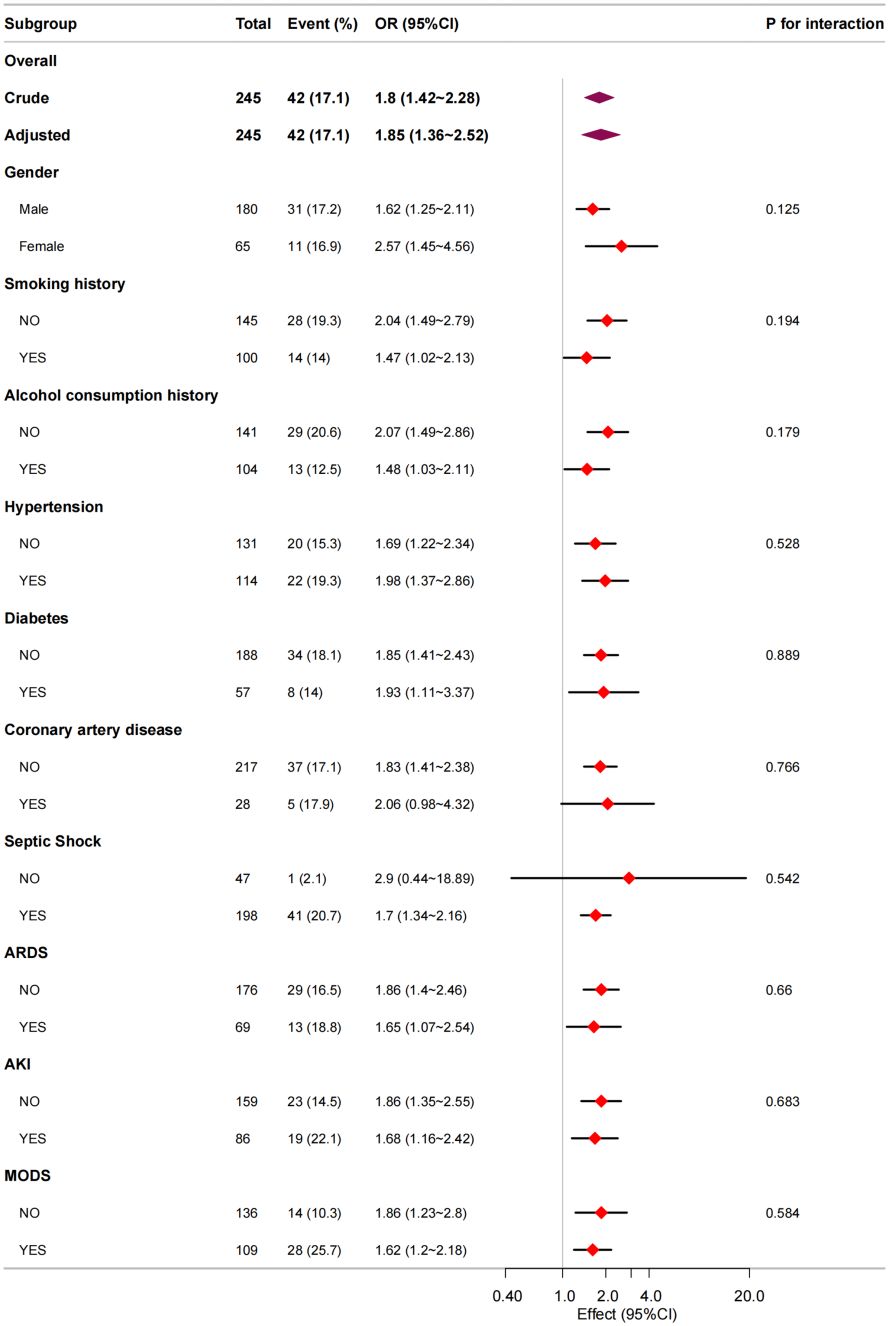
Subgroup analysis in NUTRIC score and ICU mortality.

Following covariate adjustment, we conducted stratified analyses by gender, smoking history, alcohol consumption history, comorbidities (hypertension, diabetes, coronary artery disease), and complications (septic shock, ARDS, AKI, MODS) to evaluate the consistency of the NUTRIC-ICU mortality association across different patient subgroups. As presented in Figure 3, all subgroups demonstrated robust positive associations between higher NUTRIC scores and increased ICU mortality (all adjusted ORs > 1). Notably, no significant interaction effects were observed (all P_interaction_ >0.05), indicating consistent prognostic value of NUTRIC score across all evaluated clinical strata.

### 3.5 Kaplan–Meier analysis of cumulative survival probability by NUTRIC score in ICU

**Figure 4:**
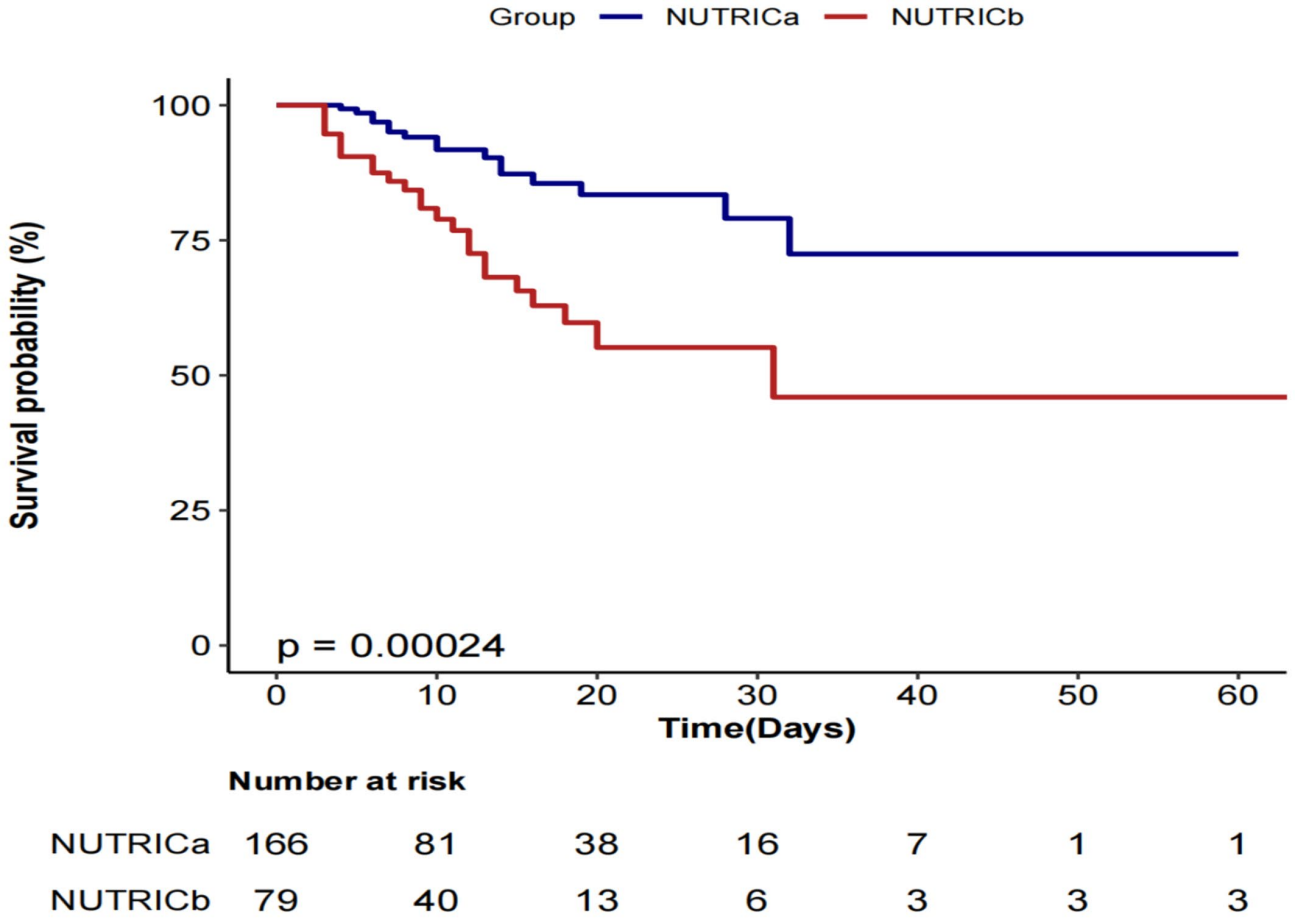
Kaplan–Meier-based survival curve.

Kaplan–Meier survival analysis revealed significantly lower cumulative survival probabilities in the high nutritional risk group (NUTRICb) compared to the low risk group (NUTRICa) (Figure 4). The log-rank test demonstrated statistically significant between-group differences (*p* = 0.00024), indicating that elevated nutritional risk was associated with increased ICU mortality risk.

## 4 Discussion

In this single-center prospective cohort study, we examined the association between the initial NUTRIC score—measured within 24 h of ICU admission—and ICU mortality in 245 patients with sepsis. The ICU mortality rate in our cohort was 17.1% (42/245). Across four multivariate logistic regression models, a significant positive association was observed between the NUTRIC score and ICU mortality. In the fully adjusted model, each one-point increase in the NUTRIC score was associated with a 92% higher risk of ICU death (OR = 1.92, 95% CI:1.32–2.80, *p* = 0.001). We also stratified patients by NUTRIC score quartiles, using the lowest quartile as the reference. A clear upward trend in ICU mortality risk was observed across increasing quartiles, and this trend remained statistically significant in all models (trend test *p* < 0.05). The RCS analysis revealed a significant linear dose–response relationship between NUTRIC score and ICU mortality (P _non-linearity_ = 0.704). Subgroup analyses across all clinical strata showed robust positive associations (all adjusted ORs > 1) without significant interaction effects (all P_interaction_ > 0.05), confirming NUTRIC score as an independent risk factor. Furthermore, Kaplan–Meier survival curves with log-rank testing (*p* = 0.00024) showed significantly lower cumulative survival in high-risk patients (NUTRICb, score ≥6) compared to low-risk patients (NUTRICa, score <6). These findings collectively establish NUTRIC score as a reliable predictor of ICU mortality in sepsis patients.

Sepsis, as a syndrome of severe organ dysfunction, imposes a substantial global disease burden and consumes significant healthcare resources. A global epidemiological study (12) reported approximately 48.9 million sepsis cases and 11 million sepsis-related deaths in 2017 alone. The mortality rate for hospitalized patients with sepsis is estimated to be around 27%, and this figure increases to approximately 42% among those admitted to the ICU (13). In addition to its high mortality, sepsis is associated with intensive utilization of medical resources. Several studies (14–16) have shown that patients with sepsis require extensive supportive care during ICU stays and that their healthcare costs within 12 months of hospitalization are significantly higher than those of patients with other conditions. Malnutrition is common among patients with sepsis and septic shock and has been closely linked to poor clinical outcomes, particularly in relation to impaired infection control and weakened immune responses (17). Given these challenges, early nutritional risk assessment may serve as a crucial step toward improving prognosis in this high-risk population. Timely identification of patients at nutritional risk and the implementation of individualized nutritional support strategies could potentially mitigate adverse outcomes in septic patients.

Although numerous tools are available for assessing nutritional status, not all are specifically designed or validated for use in critically ill patients. The Subjective Global Assessment (SGA), developed by Detsky et al. (18) in 1987, lacks objective protein-related biomarkers and relies heavily on non-quantitative, subjective measures, which may introduce measurement bias. Furthermore, SGA has limited sensitivity in detecting short-term changes in nutritional status, making it suboptimal for acute clinical settings. The NRS 2002, introduced by the ESPEN (19), was the first evidence-based screening tool for nutritional risk. However, it automatically classifies ICU patients with an APACHE II score ≥10 as high-risk, without further stratification. This lack of specificity reduces its clinical utility in the heterogeneous ICU population (20).

The NUTRIC score was developed in 2011 by Heyland and colleagues (7) in Canada as the first nutritional risk screening tool specifically designed for critically ill patients. This scoring system is based on a theoretical model of nutritional risk in critical illness, which incorporates four key domains: starvation, immune function, nutritional status, and clinical outcomes. A NUTRIC score ≥6 is generally considered indicative of high nutritional risk. Because IL-6, one of the original components of the score, is not routinely measured in ICU settings, Rahman et al. (21) proposed a modified version—the mNUTRIC score—which excludes IL-6. In the mNUTRIC system, a score ≥5 denotes high nutritional risk. Importantly, Jeong et al. (22) demonstrated that the predictive accuracy of the tool remained unchanged after the exclusion of IL-6.

Compared with other nutritional screening tools, the NUTRIC score uniquely integrates indicators of disease severity, including the APACHE II and SOFA scores. This helps address the limitations of general screening tools that often omit inflammation-related parameters. Furthermore, the NUTRIC score is suitable for patients with impaired consciousness or those on mechanical ventilation, as it does not rely on subjective communication. It has demonstrated strong predictive value for malnutrition risk and clinical outcomes in critically ill adult patients and is now widely used in clinical practice as a reliable tool for nutritional risk assessment in the ICU.

The NUTRIC score has been widely applied in prognostic research among critically ill patients. Zhang et al. (23) evaluated nutritional risk in patients admitted to a neuro-intensive care unit (NICU) using NRS 2002, NUTRIC, and mNUTRIC scores. They compared the predictive value of NUTRIC and mNUTRIC scores for 28-day mortality, reporting AUCs of 0.857 (95% CI: 0.786–0.928) and 0.856 (95% CI: 0.786–0.927), respectively. Their results indicated that the mNUTRIC score was independently associated with 28-day mortality and is a suitable tool for both nutritional risk screening and prognosis prediction in NICU patients. Canales et al. (24) further demonstrated a significant association between higher NUTRIC scores and inadequate micronutrient delivery in ICU patients. Specifically, each one-point increase in the NUTRIC score was associated with an additional protein deficit of 52 grams and an energy deficit of 3,385 kJ. High-risk patients had a 2.87-fold greater risk of cumulative protein deficit (≥300 g) and a 3.10-fold greater risk of cumulative energy deficit (≥25,104 kJ) compared to low-risk patients (both *p* < 0.001). Compher et al. (25) reported that critically ill patients with higher NUTRIC scores and longer ICU stays benefited more from adequate protein and energy delivery. Among high-risk patients, achieving nutritional targets was associated with significantly lower 60-day mortality compared to those who did not meet their nutritional requirements. In a prospective observational study (26) from Iran involving 1,311 ICU patients, NRS 2002, SGA, and NUTRIC scores were compared, with one tool used as the reference standard to evaluate the predictive validity of the others. The NUTRIC score demonstrated the highest diagnostic accuracy for malnutrition, with an AUC of 0.74.

Given that IL-6 is not routinely measured in ICU settings, recent studies have increasingly adopted the mNUTRIC score to predict outcomes in patients with sepsis. A Korean cohort study (22) involving 482 patients with sepsis compared the predictive performance of the NUTRIC and mNUTRIC scores for 28-day mortality. The AUCs were 0.762 (95% CI: 0.718–0.806) for the NUTRIC score and 0.757 (95% CI: 0.713–0.801) for the mNUTRIC score, with no significant difference between the two (*p* = 0.45). Wełna et al. (9) applied the mNUTRIC score upon ICU admission to assess nutritional status in sepsis patients. They reported an AUC of 0.833 (95% CI: 0.76–0.89, *p* < 0.001) for predicting 28-day mortality. Patients with mNUTRIC scores ≥6 were more likely to exhibit higher mortality, greater ICU resource utilization, increased demand for corticosteroids and blood products, and a higher nursing workload.

Although these studies demonstrate that both NUTRIC and mNUTRIC scores are effective in predicting prognosis in sepsis patients, we argue that IL-6 should not be entirely excluded from nutritional risk assessment. According to a prospective cohort study (27), early measurement of serum IL-6 levels can indicate disease severity in sepsis, regardless of immune status. Therefore, the prognostic value of NUTRIC versus mNUTRIC in sepsis warrants further investigation, particularly regarding the potential role of IL-6 in enhancing risk stratification and clinical decision-making.

The NUTRIC score incorporates objective parameters that are readily available in the ICU setting and accommodates the specific challenges of critically ill patients, such as impaired consciousness and prolonged immobility. It has demonstrated strong predictive value for both malnutrition risk and mortality, making it a promising tool in guiding nutritional support strategies in the ICU. Given its clinical utility in identifying patients at nutritional risk, we recommend that the NUTRIC score be applied as early as possible following ICU admission. Early assessment enables timely identification of high-risk individuals, allowing for closer monitoring and systematic implementation of individualized nutritional support, which may ultimately reduce mortality and improve overall patient outcomes.

Although our study demonstrated a significant positive association between the NUTRIC score and ICU mortality in patients with sepsis, with subgroup analyses confirming the consistency of this relationship across gender, smoking history, alcohol consumption history, medical history, and complications, several limitations warrant consideration. First, IL-6—a key component of the original NUTRIC score—is not routinely measured in most ICU settings, which may limit the feasibility of its widespread clinical application due to difficulties in parameter acquisition. Second, as a single-center, disease-specific, and small-sample prospective study, our findings may be subject to selection bias and should be cautiously extrapolated to other critically ill populations; the predominance of male patients (73.5%) and the exclusion of pregnant women further restrict generalizability to females. Therefore, future research should involve multicenter, large-scale prospective studies—including pregnant and non-pregnant women—to externally validate the robustness of the NUTRIC score in predicting ICU mortality and to confirm its external validity in sepsis. In addition, randomized controlled trials are needed to investigate the quantitative relationship between NUTRIC-based nutritional risk stratification and clinical outcomes. These studies should also evaluate the impact of individualized nutritional interventions on mortality across different risk levels. Ultimately, such efforts may contribute to the development of a personalized, NUTRIC-guided nutritional support strategy that enables dynamic and precise nutritional management in critically ill patients, thereby optimizing clinical outcomes and improving patient benefit.

## 5 Conclusion

Our findings indicate a linear positive correlation between NUTRIC score and ICU mortality risk in sepsis patients. Further analysis revealed that the impact of NUTRIC score on ICU mortality was not modified by factors such as gender, smoking history, alcohol consumption history, medical history, or comorbidities, suggesting its strong general applicability as an independent risk assessment indicator. Based on these findings, we recommend adopting the NUTRIC score as a routine nutritional assessment tool for sepsis patients upon ICU admission to facilitate early identification of high mortality risk patients. Additionally, clinical healthcare providers may combine the NUTRIC score with other clinical indicators to develop personalized intervention plans, which may potentially improve the prognosis of sepsis patients in the ICU.

## Data Availability

The raw data supporting the conclusions of this article will be made available by the authors, without undue reservation.
